# Five‐year implant survival does not differ between hybrid and cementless total knee arthroplasty in a cohort of 5361 patients using a deep‐dish mobile bearing design

**DOI:** 10.1002/ksa.70075

**Published:** 2025-09-30

**Authors:** Ophélie Manchec, Emilie Bérard, Alessandro Carrozzo, Regis Pailhé, Etienne Cavaignac

**Affiliations:** ^1^ Service de Chirurgie Orthopédique et Traumatologie, Hôpital Pierre‐Paul Riquet, CHU Purpan Toulouse France; ^2^ Service d'Épidémiologie Clinique et de Santé Publique, CHU de Toulouse, CERPOP, Inserm Université de Toulouse III Paul Sabatier Toulouse France; ^3^ Link Campus University Rome Italy; ^4^ Service de Chirurgie Orthopédique, Clinique Aguiléra, Ramsay Santé Biarritz France

**Keywords:** cementless, hybrid, mobile bearing, prothesis design, survival rate, total knee arthroplasty

## Abstract

**Purpose:**

This study compared total knee arthroplasty (TKA) outcomes between cementless and hybrid fixation, with separate analysis of tibial and femoral cementation. The primary hypothesis was that equivalence would exist in implant survivorship between cementless and hybrid fixation techniques. The secondary hypothesis was that cementless and hybrid TKAs would demonstrate comparable mid‐term surgery‐free survival rates, rates of aseptic reoperation and functional outcomes.

**Methods:**

A multicentre retrospective study was conducted using prospectively collected data from a large cohort, all implanted with the same deep‐dish mobile bearing. Patients were divided according to the fixation method: fully cementless, femoral cemented/tibial uncemented and tibial cemented/femoral uncemented. The survival rates of cementless and hybrid TKA were compared with a median follow‐up of 27 months. Evolution of functional outcomes (International Knee Society [IKS] score, range of motion [ROM]) from preoperative to 5‐year follow‐up were also compared. Propensity score matching (PSM) was performed to balance covariates (age, sex, body mass index and patellar procedure) between groups.

**Results:**

Among the 5361 primary TKA included (4549 cementless, 435 tibial hybrid and 377 femoral hybrid), 1505 reached the 5‐year follow‐up. Five‐year revision‐free survival rate was 98.7% (95% confidence interval: 98.2; 99.1) for cementless, 97.8% (94.6; 99.1) for tibial hybrid (*p* = 0.537) and 98.4% (94.9; 99.5) for femoral hybrid (*p* = 0.669). Cumulative surgery‐free survival rates and functional outcomes at 5 years showed no significant differences between groups with clinically comparable results.

**Conclusion:**

No difference in survivorship was observed between cementless and hybrid TKA at 5 years in this cohort of 5361 patients, whether the tibia or femur was cemented. Rates of reoperation and aseptic reoperation were also comparable, as were clinical outcomes. These results suggest that the choice between hybrid and cementless fixation does not impact mid‐term TKA survival.

**Level of Evidence:**

Level III.

**Abbreviations**
ASA: American Society of Anaesthesiologists.
BMI: body mass index
C: cemented
CI: confidence interval
HKA: hip knee angle
IKS: international knee score
mFA: mechanical femoral axis
mTA: mechanical tibial axis
NC: cementless
PCL: posterior cruciate ligament
PSM: propensity score matching
RCT: randomized controlled trial
ROM: range of motion
TKA: total knee arthroplasty

## INTRODUCTION

As total knee arthroplasty (TKA) remains one of the most commonly performed orthopaedic procedures [11, 14], optimising its long‐term survivorship is a key objective. Among the factors influencing implant longevity, the method of fixation continues to be debated, especially with the great results of second‐generation cementless designs [1, 3, 5, 9]. While numerous studies have compared fully cemented and fully cementless TKAs [8, 17, 21, 24], evidence on hybrid fixation, where only the femur or tibia is cemented, remains limited and inconsistent. The heterogeneity in study designs, implant generations and definitions of hybrid fixation (e.g., tibial‐only vs. femoral‐only cementation) complicates interpretation [10, 12, 22]. Moreover, the femur and tibia differ biomechanically, with the tibia mainly subjected to compression and shear forces, while the femur endures more flexion‐extension and rotational stresses due to its convex anatomy and broader fixation surface, raising the question of whether fixation site affects implant survival. This distinction has practical implications in surgical decision‐making, as cemented fixation may be favoured on the tibial side to counteract the higher risk of micromotion under load, whereas the femoral side, with its larger contact area and better vascularised bone, may be more amenable to durable biological fixation [16, 18, 19].

The aim of this study was to assess the impact of hybrid fixation on TKA outcomes by comparing cementless to hybrid TKAs, with separate analysis of tibial and femoral cementation, using the same implant design.

Our primary hypothesis was that there would be no difference in revision‐free survival rate between cementless and hybrid TKA, whether the femur or the tibia is cemented. Our secondary hypothesis was that there would be no difference in reoperation, even with septic cases excluded, or functional outcomes at 5‐year follow‐up.

## METHODS

This multicentre retrospective cohort study is based on prospectively collected data from the Amplitude® industrial arthroplasty registry (Valence, France), including all the patients who underwent TKA using the SCORE I implant for primary osteoarthritis. The registry was utilized under authorization from the French data protection authority (CNIL), registered on CliniRecord under number 1355265, and declared on the public Health Data Hub platform under number F20210913151920. Data management complied with the CNIL's MR‐004 reference methodology. Surgical procedures were conducted across 15 French healthcare centres by 16 different orthopaedic surgeons.

The SCORE I implant (Amplitude®) is a fully congruent TKA, posterior cruciate ligament sacrificing, featuring a mobile bearing and rotating platform. Both cemented and cementless versions have the same design. For cementless fixation, the implants are coated with dual 80 µm layers of plasma‐sprayed titanium and hydroxyapatite.

All patients who received a primary SCORE I TKA between March 2002 and July 2022 were considered for inclusion. Exclusion criteria included missing cementation data, TKA for inflammatory arthritis, the use of reconstruction implants (mainly for oncologic indications), and cases with fully cemented fixation. The cohort was divided into three groups based on fixation method: entirely cementless implants; hybrid fixation with a cemented femoral component and an uncemented tibial component; and hybrid fixation with a cemented tibial component and an uncemented femoral component. Patellar component cementation was not considered in group allocation.

Preoperative and postoperative data were collected prospectively. Primary outcome was implant survival defined as the time elapsed (in months) between the date of surgery and the first revision procedure involving a partial or total change or removal of the implant, regardless of the cause (e.g., loosening, patellar complications, infection, fracture, stiffness, pain or dislocation). Patients without revision were censored at the date of their last follow‐up or death.

Secondary outcomes included surgery‐free survival, defined as any reoperation on the operated knee, regardless of whether the implant was revised. Another endpoint was aseptic surgery‐free survival, where events were defined as any reoperation on the operated knee excluding those performed for infection.

The International Knee Society (IKS) score [20] was recorded preoperatively and at subsequent clinical follow‐ups, with only the 5‐year data used for analysis. The knee's flexion range [15], in degrees, was also measured before, during, and after the procedure, and the 5‐year values were used for comparison.

Standard radiographic imaging of the knee was performed. Angular measurements including the hip‐knee‐ankle (HKA) angle, mechanical femoral angle (mFA) and mechanical tibial angle (mTA) were manually assessed in degrees by the surgeon using dedicated imaging software, both before surgery and 1 year postoperatively.

### Statistical analysis

For the primary endpoint, a sample size of 4549 cementless TKA, 435 hybrid cemented tibial TKA and 377 hybrid cemented femoral TKA achieved 80% power to detect equivalence with a margin of ±3%, a reference group proportion expected to 97% and an actual difference fixed to 0. The significance level is 0.025 (using the Bonferroni correction for two comparisons: cementless vs. hybrid cemented tibial TKA and cementless vs. hybrid cemented femoral TKA).

Before analyses, verification of missing or aberrant or inconsistent data were conducted. After corrections, the database was locked. Analysis was performed on the locked database.

Characteristics of patients were first described using the appropriate descriptive statistics according to the type of variables. Descriptive statistics included mean with standard deviation (SD) and range (minimum–maximum), for continuous variables, and number of nonmissing observation with frequency (%) for categorical variables. Then, categorical variables were compared between groups using the *χ* ^2^‐test (or Fisher's exact test when necessary). Student's *t*‐test was used to compare the distribution of continuous variables (or Mann–Whitney's test when distribution departed from normality or when homoscedasticity is rejected).

For the analysis of the survival endpoints, Kaplan–Meier survival curves were described together with 95% confidence interval (CI) and compared, first using the log‐rank test, and then using a cox model adjusted for age, sex, patellar procedure, body mass index (BMI) and period (2002–2011 vs. 2012–2022).

Finally, as a sensitivity analysis, the propensity score method was used to more extensively take into account potential baseline differences between hybrid tibial (or femoral) cemented TKA versus cementless TKA. A multivariate logistic regression model was generated to estimate for each patient a propensity score to receive hybrid tibial (or femoral) cemented TKA versus cementless TKA. Covariates were period of intervention, age, sex, BMI, ASA (American Society of Anaesthesiologists) score, approach, tourniquet, navigation, patellar procedure and patellar type. The performance of the model was appreciated with the *c*‐statistic (0.74 [95% CI: 0.72–0.77] for hybrid tibial cemented TKA vs. cementless TKA and 0.83 [0.81–0.86] for hybrid femoral cemented TKA vs. cementless TKA). Mean propensity score, for hybrid tibial cemented TKA versus cementless TKA, was 0.152 (±0.100) in patients with hybrid tibial cemented TKA (*N* = 435) and 0.082 (±0.070) in patients with cementless TKA (*N* = 4528). Mean propensity score, for hybrid femoral cemented TKA versus cementless TKA, was 0.315 (±0.249) in patients with hybrid femoral cemented TKA (*N* = 377) and 0.057 (±0.087) in patients with cementless TKA (*N* = 4528). According to propensity score, 421 patients with hybrid tibial cemented TKA were matched with 421 cementless TKA (596 with a precision of 0.0001, 40 with a precision of 0.001, 138 with a precision of 0.01 and 68 with a precision of 0.1) and 285 patients with hybrid femoral cemented TKA were matched with 285 cementless TKA (464 with a precision of 0.0001, 24 with a precision of 0.001, 28 with a precision of 0.01 and 54 with a precision of 0.1). Mean propensity score was the same in patients with hybrid tibial or femoral cemented and cementless TKA in the matched samples (0.141 ± 0.082 and 0.227 ± 0.218, respectively). Survival rate before revision, surgery‐free survival rate and surgery‐free survival rate without sepsis were compared between cemented and cementless TKA in the subgroups of propensity score matched subjects.

Student's *t*‐test (or Mann–Whitney's test if necessary) was used to compare the distribution of continuous secondary endpoints. The evolution of IKS and flexion at 5 year was also compared between groups after adjustment for preoperative value, age, sex, patellar procedure, BMI and period, using a linear regression model.

All reported *p*‐values were two‐sided and the significance threshold was <0.025 (using the Bonferroni correction for two comparisons: cementless vs. hybrid cemented tibial TKA and cementless vs. hybrid cemented femoral TKA).

Statistical analyses were performed using STATA software 18.0 (STATA Corp).

## RESULTS

The survival analysis included 5361 TKAs, of which 4549 (84.9%) were in the cementless group and 812 (15.1%) in the hybrid group, including 435 cases with tibial cementation (8.1%) and 377 with femoral cementation (7.0%). A total of 1505 patients reached the 5‐year follow‐up mark. For the secondary endpoints analysis, focusing on functional outcomes, 424 patients with complete 5‐year follow‐up data were included.

**Figure 1:**
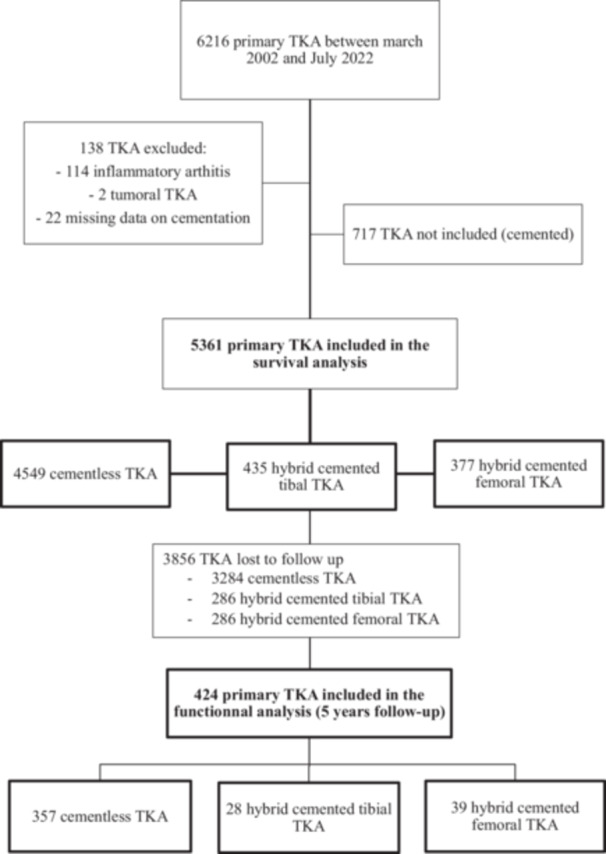
Flowchart of the inclusion of the patients. TKA, total knee arthroplasty.

The inclusion process is outlined in the corresponding flowchart (Figure 1). Baseline patient characteristics are presented in Table 1, while pre‐ and postoperative radiographic alignment measurements are detailed in Table 2. Analyses were adjusted for the main variables reported in the literature to influence implant survival. It should be noted that the groups also differed in the use of a tourniquet, navigation and patellar component type. These factors were not included in the adjustment model, as their impact on mid‐term survivorship is not consistently supported by previous studies.

**Table 1:** Patients characteristics.

Characteristics	Cementless TKA (*n* = 4549)	Cemented tibial TKA (*n* = 435)	*p* value	Cemented femoral TKA (*n* = 377)	*p* value
Intervention period *n* (%)			<0.0001		<0.0001
2002–2011	2113 (46.4)	339 (77.9)		315 (83.6)	
2012–2022	2436 (53.6)	96 (22.1)		62 (16.4)	
Sex *n* (%)			0.010		0.001
Men	1772 (40.0)	141 (33.7)		117 (31.3)	
Women	2659 (60.0)	278 (66.3)		257 (68.7)	
Age (years)			0.334		<0.0001
*n*/missing	4474/75	427/8		372/5	
Mean (SD)	71 (9.0)	71 (9.6)		73 (8.4)	
Min; max	18; 94	27; 90		47; 90	
Side *n* (%)			0.203		0.233
Left	2184 (48.0)	195 (44.8)		169 (44.8)	
Right	2365 (52.0)	240 (55.2)		208 (55.2)	
Weight (kg)			<0.0001		<0.0001
*n*/missing	4243/306	411/24		357/20	
Mean (SD)	82.0 (15.6)	77.8 (14.2)		78.8 (15.5)	
Min; max	42; 160	45; 141		44; 150	
BMI (kg/m^2^)			<0.0001		0.051
*n*/missing	4212/337	411/24		355/22	
Mean (SD)	30.26 (5.33)	29.1 (5.0)		29.7 (5.7)	
Min; max	16.00; 55.00	17.0; 47.0		21.0; 55.0	
ASA *n* (%)			0.270		<0.0001
1	416 (10.6)	53 (13.5)		32 (8.9)	
2	2396 (60.8)	235 (59.6)		271 (75.5)	
3	1105 (28.1)	106 (26.9)		56 (15.6)	
4	20 (0.5)	0 (0.0)		0 (0.0)	
Approach *n* (%)			0.107		0.796
Anteromedial	3843 (91.4)	325 (93.9)		325 (91.0)	
Anterolateral	360 (8.6)	21 (6.1)		32 (9.0)	
Tourniquet *n* (%)			<0.0001		0.007
Yes	252 (7.1)	1 (0.3)		3 (1.8)	
No	3274 (92.9)	287 (99.7)		165 (98.2)	
Navigation *n* (%)			0.001		<0.0001
Yes	2537 (55.8)	278 (63.9)		311 (82.5)	
No	2012 (44.2)	157 (36.1)		66 (17.5)	
Patellar procedure *n* (%)			<0.0001		<0.0001
Yes	1637 (36.0)	207 (47.6)		252 (66.8)	
No	2912 (64.0)	228 (52.4)		125 (33.2)	
Patellar type *n* (%)			<0.0001		<0.0001
Cementless inlay patella	476 (29.1)	27 (13.0)		13 (5.2)	
Cemented inlay patella	489 (29.9)	73 (35.3)		12 (4.8)	
Resurfaced cemented patella	672 (41.1)	107 (51.7)		227 (90.1)	

*Note*: The significance threshold was set at <0.025, applying the Bonferroni correction for two comparisons (cementless vs. hybrid tibial cemented TKA and cementless vs. hybrid femoral cemented TKA).

Abbreviations: ASA, American Society of Anaesthesiologists; BMI, body mass index; Max, maximum; Min, minimum; SD, standard deviation.

**Table 2:** Radiological measurements before and 1 year after surgery.

Radiological measurements	Cementless TKA	Cemented Tibial TKA	*p* value	Cemented femoral TKA	*p* value
Preoperative HKA (°)			<0.0001		0.235
*n*/missing	3261/1288	285/150		259/118	
Mean (SD)	177.0 (5.2)	178.8 (5.7)		177.1 (5.5)	
Postoperative HKA (°)			0.787		0.785
*n*/missing	1691/2858	136/299		144/233	
Mean (SD)	179.3 (2.3)	179.4 (2.4)		179.3 (2.1)	
Preoperative mFA (°)			0.103		<0.0001
*n*/missing	2924/1625	280/155		260/117	
Mean (SD)	91.3 (2.9)	91.6 (3.9)		94.5 (4.0)	
Postoperative mFA (°)			0.024		<0.0001
*n*/missing	926/3623	52/383		53/324	
Mean (SD)	90.3 (1.8)	90.9 (2.6)		94.0 (3.6)	
Preoperative mTA (°)			<0.0001		0.210
*n*/missing	2631/1918	258/177		106/271	
Mean (SD)	87.2 (3.8)	88.8 (5.5)		87.7 (4.8)	
Postoperative mTA (°)			0.143		0.372
*n*/missing	776/3773	39/396		21/356	
Mean (SD)	89.8 (1.6)	90.2 (2.3)		89.5 (1.5)	

*Note*: The significance threshold was set at <0.025, applying the Bonferroni correction for two comparisons (cementless vs. hybrid tibial cemented TKA and cementless vs. hybrid femoral cemented TKA).

Abbreviations: HKA, hip knee angle; mFA, mechanical femoral angle; mTA, mechanical tibial angle; SD, standard deviation; TKA, total knee arthroplasty.

### Primary endpoint—Survival rate

**Figure 2:**
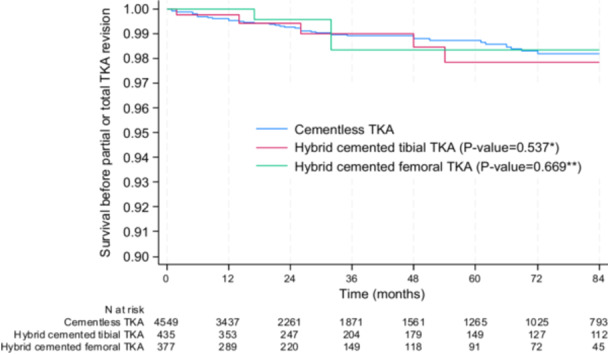
Revision free‐survival rate between cementless, hybrid cemented tibial TKA and hybrid cemented femoral TKA. *p value for the comparison between cementless TKA and hybrid TKA with cemented tibia; **p value for the comparison between cementless TKA and hybrid TKA with cemented femur; the significance threshold was set at <0.025, applying the Bonferroni correction for two comparisons (cementless vs. hybrid tibial cemented TKA and cementless vs. hybrid femoral cemented TKA). TKA, total knee arthroplasty.

Overall, the cumulative survival rate between cementless, hybrid cemented tibial and hybrid cemented femoral TKA was not statistically different (Figure 2). At 5 years, the survival rate before revision was 98.7% (CI: 98.2–99.1) in the cementless group, 97.8% (CI: 94.6–99.1) in the hybrid cemented tibial group (*p* = 0.537) and 98.4% (CI: 94.9–99.5) in the hybrid cemented femur group (*p* = 0.669). The ±3% equivalence margin was not reached.

Results were the same when adjusted for age, sex, BMI, patellar procedure and period (2002–2011 vs. 2012–2022) (P Cox‐model = 0.636, for cemented tibia vs. cementless TKA and P Cox‐model = 0.574, for cemented femur vs. cementless TKA) and in the sensitivity analysis conducted in the subgroups of propensity score matched subjects. Indeed, at 5 years, the survival rate before revision was 97.8% [CI: 95.0–99.0] in the matched cementless TKA group and 97.8%, [CI: 94.5–99.1] in the matched hybrid tibial cemented TKA group (*p* = 0.945). The 5‐years survival rate before revision was 98.4% [CI: 95.7–99.4] in the matched cementless TKA group and 98.3%, [CI: 94.8–99.5] in the matched hybrid femoral cemented TKA group (*p* = 0.777).

**Figure 3:**
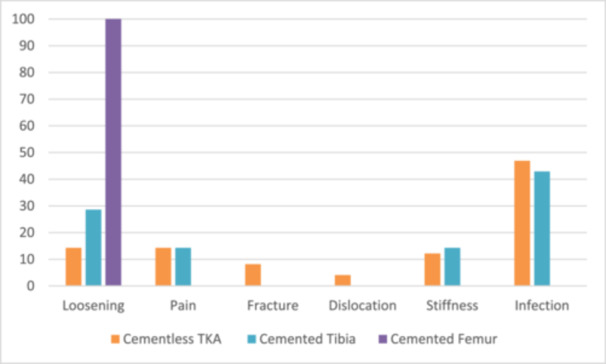
Distribution of causes of revision by fixation type.

The various reasons for revision are listed in Table 3 and the distribution by type of fixation in Figure 3. The primary reason for revision was infection in the cementless (46.9%) and cemented tibia group (42.9%) and loosening in cemented femur group (100%) (*p* = 0.967, for cemented tibia vs. cementless TKA and *p* = 0.052, for cemented femur vs. cementless TKA). It was followed by loosening (14.3% in the cementless group and 28.6% in the cemented tibia group) and pain in the cementless group (14.3%). The relatively small number of revision events (*n* = 59) limits the robustness of subgroup analyses regarding reasons for failure, and these findings should therefore be interpreted with caution.

**Table 3:** Reason for revision by type of cementation.

	Cementless TKA	Cemented tibia	*p* value	Cemented femur	*p* value	Total
	*n* = 49 (83.1%)	*n* = 7 (11.9%)		*n* = 3 (5.1%)		*n* = 59 (100%)
Cause *n* (%)			0.967		0.052	
Loosening	7 (14.3)	2 (28.6)		3 (100.0)		12 (20.3)
Pain	7 (14.3)	1 (14.3)		0 (0.0)		8 (13.6)
Fracture	4 (8.2)	0 (0.0)		0 (0.0)		4 (6.8)
Dislocation	2 (4.1)	0 (0.0)		0 (0.0)		2 (3.4)
Stiffness	6 (12.2)	1 (14.3)		0 (0.0)		7 (11.9)
Infection	23 (46.9)	3 (42.9)		0 (0.0)		26 (44.1)

*Note*: The significance threshold was set at <0.025, applying the Bonferroni correction for two comparisons (cementless vs. hybrid tibial cemented TKA and cementless vs. hybrid femoral cemented TKA).

Abbreviation: TKA, total knee arthroplasty.

### Secondary endpoints—Surgery‐free survival rate, surgery‐free survival rate without infection and functional outcomes

**Figure 4:**
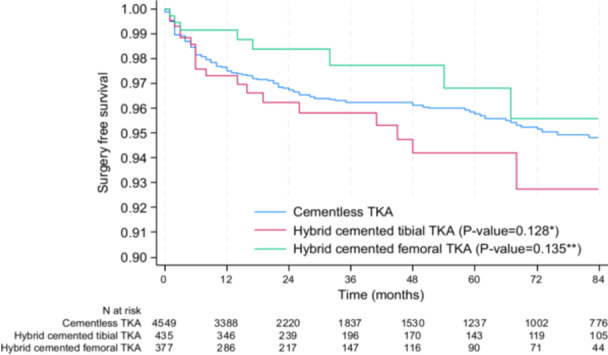
Surgery‐free survival rate between cementless, hybrid cemented tibial TKA and hybrid cemented femoral TKA. *p value for the comparison between cementless TKA and hybrid TKA with cemented tibia; **p value for the comparison between cementless TKA and hybrid TKA with cemented femur; the significance threshold is <0.025 (using the Bonferroni correction for two comparisons: cementless vs. hybrid cemented tibial TKA and cementless vs. hybrid cemented femoral TKA). TKA, total knee arthroplasty.

The cumulative surgery‐free survival rate was not statistically different between cementless, hybrid cemented tibial and hybrid cemented femoral TKA (Figure 4), with a survival rate at 5 years of 95.8% (CI: 94.9–96.5) in the cementless group, 94.2% (CI: 90.7–96.4) in the hybrid cemented tibial group (*p* = 0.128) and 96.8% (CI: 92.9–98.6) in the hybrid cemented femur group (*p* = 0.135).

Results were the same when adjusted for age, sex, BMI, patellar procedure and period (2002–2011 vs. 2012–2022) (P Cox‐model = 0.103, for cemented tibia vs. cementless TKA and P Cox‐model = 0.245, for cemented femur vs. cementless TKA) and in the sensitivity analysis conducted in the subgroups of propensity score matched subjects. Indeed, at 5 years, the surgery‐free survival rate was 94.7% (CI: 91.6–96.7) in the matched cementless TKA group and 94.3% (CI: 90.6–96.5) in the matched hybrid tibial cemented TKA group (*p* = 0.546).

The 5‐year surgery‐free survival rate was 95.6% (CI: 92.6–97.4) in the matched cementless TKA group and 95.3% (CI: 91.7–97.3) in the matched hybrid femoral cemented TKA group (*p* = 0.755).

**Figure 5:**
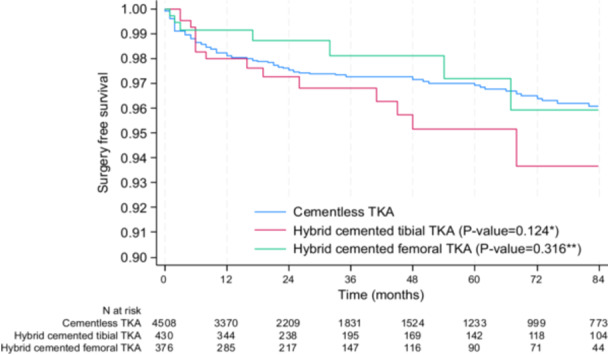
Surgery‐free survival rate between cementless, hybrid tibial cemented and hybrid femoral cemented TKA, infections excluded (*N* = 47). *p value for the comparison between cementless TKA and hybrid TKA with cemented tibia; **p value for the comparison between cementless TKA and hybrid TKA with cemented femur; the significance threshold is <0.025 (using the Bonferroni correction for two comparisons: cementless vs. hybrid cemented tibial TKA and cementless vs. hybrid cemented femoral TKA). TKA, total knee arthroplasty.

After all patients who underwent surgery for infection were excluded (*N* = 47), the cumulative surgery‐free survival rate was not statistically different between cementless, hybrid cemented tibial and hybrid cemented femoral TKA (Figure 5), with a survival rate at 5 years of 96.9% (CI: 96.2–97.5) in the cementless group, 95.2% (CI: 91.7–97.2) in the hybrid cemented tibial group (*p* = 0.124) and 97.2% (CI: 93.2–98.9) in the hybrid cemented femur group (*p* = 0.316).

Results were the same when adjusted for age, sex, BMI, patellar procedure and period (2002–2011 vs. 2012–2022) (P Cox‐model = 0.140, for cemented tibia vs. cementless TKA and P Cox‐model = 0.471, for cemented femur vs. cementless TKA) and in the sensitivity analysis conducted in the subgroups of propensity score matched subjects. Indeed, at 5 years, the surgery‐free survival rate, sepsis excluded, was 95.6% (CI: 92.6–97.4) in the matched cementless TKA group and 95.3% (CI: 91.7–97.3) in the matched hybrid tibial cemented TKA group (*p* = 0.755).

The 5‐year surgery‐free survival rate, sepsis excluded, was 95.6% (CI: 92.6–97.4) in the matched cementless TKA group and 95.3% (CI: 91.7–97.3) in the matched hybrid femoral cemented TKA group (*p* = 0.755).

There was no significant difference in the improvement in functional outcomes at 5 years (Table 4). The 5‐year IKS score was 179 (±25) in the cemented tibial TKA group, reflecting an improvement of 76 points (±31) from the preoperative score (*p* = 0.121), and 182 (±25) in the cemented femoral TKA group, reflecting an improvement of 87 points (±38) from the preoperative score (*p* = 0.692). Results were the same when adjusted for preoperative IKS, age, sex, patellar procedure, BMI and period (2002–2011 vs. 2012–2022) (*p* = 0.556, for cemented tibia vs. cementless TKA and *p* = 0.731, for cemented femur vs. cementless TKA).

**Table 4:** Functional outcomes before surgery and at 5 years [20].

Measure	Cementless TKA	Cemented tibial TKA	*p* value	Cemented femoral TKA	*p* value
Preoperative flexion (°)			<0.0001		<0.0001
*n*/missing	4316/233	416/19		371/6	
Mean (SD)	113 (14)	111 (15)		109 (14)	
Flexion at 5 years (°)			0.345		0.779
*n*/missing	357/4192	29/406		40/337	
Mean (SD)	118 (11)	116 (11)		119 (13)	
Change in flexion at 5 years (post–preoperative)			0.008		0.196
*n*/missing	337/4212	28/407		40/337	
Mean (SD)	5 (13)	12 (20)		8 (14)	
Preoperative IKS			0.188		0.093
*n*/missing	3640/909	345/90		285/92	
Mean (SD)	97 (21)	99 (23)		95 (22.0)	
IKS at 5 years			0.899		0.718
*n*/missing	279/4270	22/413		31/346	
Mean (SD)	182 (19)	178 (25)		182 (25)	
Change in IKS at 5 years (post–preoperative)			0.121		0.692
*n*/missing	243/4306	22/413		28/349	
Mean (SD)	84 (24)	76 (30)		87 (38)	

*Note*: The significance threshold was set at <0.025, applying the Bonferroni correction for two comparisons (cementless vs. hybrid tibial cemented TKA and cementless vs. hybrid femoral cemented TKA).

Abbreviations: IKS, international knee score; SD, standard deviation.

Five‐year knee flexion was measured at 116° (±11) in the cemented tibial TKA group, with an improvement of 12° (±20) from the preoperative flexion (*p* = 0.008), and 119° (±13) in the cemented femoral TKA group, showing an improvement of 8° (±14) from the preoperative flexion (*p* = 0.196). These results became nonsignificant in the cemented tibial TKA group when adjusted for preoperative flexion, age, sex, patellar procedure, BMI and period (2002–2011 vs. 2012–2022) (*p* = 0.603). In the cemented femoral TKA group, results were the same when adjusted for preoperative flexion, age, sex, patellar procedure, BMI and period (2002–2011 vs. 2012–2022) (*p* = 0.112).

## DISCUSSION

A key finding of this study is that the survivorship at 5 years of hybrid TKA, whether cemented on the tibia or femur, is comparable to that of fully cementless implants, within a margin of equivalence of ±3% and no statistically significant differences observed. These findings are reinforced by multivariate adjustments and propensity score matching (PSM), suggesting that partial cementation, regardless of its location, does not significantly impact mid‐term implant survivorship. They are consistent with the existing literature, which reports excellent outcomes for hybrid TKA, often in comparison to the current gold standard of fully cemented fixation. What sets our study apart is the inclusion of both tibial and femoral hybrid fixations, directly compared to a fully cementless group within a single analysis, an approach not previously reported. This allows for a more nuanced understanding of how partial cementation, depending on its location, may or may not influence implant survival. As an example, in a 10‐year randomized controlled trial (RCT), Batailler et al. reported no significant difference in survival between hybrid tibial cementation and fully cemented TKA [2]. Similarly, in a recent meta‐analysis, Wang et al. found no significant differences in mid‐term outcomes regarding aseptic loosening, reoperation rates, infection, radiolucent lines, or operating time across hybrid, cementless and cemented fixation methods [23].

Hybrid cementation is more frequently reported with tibial fixation than femoral, largely due to the higher risk of aseptic loosening or implant migration observed in early cementless designs [4, 7], which historically led to the increased adoption of hybrid fixation. However, in their randomized clinical trial, Gibon et al. reported a 10‐year revision‐free survival rate of 96% in both cementless or hybrid cemented tibial TKA [6], which is consistent with our findings. This may help to re‐evaluate the perceived limitations of cementless tibial implants and support their continued use within hybrid configurations. With the excellent results reported in the literature for cementless designs [8, 13, 21], it could be hypothesized that the choice of fixation method might be guided by the surgeon's preference and intraoperative judgment, without necessarily compromising implant durability at mid‐term follow‐up.

Surgery‐free survival and aseptic surgery‐free survival at 5 years were also similar between the two hybrid subgroups and fully cementless implants. These findings support the notion that the incidence of complications, particularly infection and aseptic loosening, is low and comparable between fixation strategies, reinforcing the idea that the fixation method may not be the primary determinant of implant durability today.

Functional outcomes, including the IKS score and flexion, improved in both hybrid groups. While femoral cementation showed a trend towards greater IKS improvement (+87), and tibial cementation was associated with slightly better gain in flexion (+12°), both not clinically relevant, neither difference reached statistical significance after adjustment when compared to fully cementless fixation. In their case‐control study of 124 patients, Lizaur‐Utrilla et al. found better clinical scores with hybrid cementless femoral than fully cemented TKA after a minimum follow‐up of 15 years [12], but these results were not clinically relevant. These trends mirror the results reported in other recent studies, which have not established a clear functional superiority of hybrid technique over fully cemented or cementless implants.

This study benefits from a large patient cohort and robust statistical methodology, including both multivariate adjustment and PSM. To our knowledge, it is the only study comparing, within a single analysis, cementless, hybrid tibial and hybrid femoral fixation using the same implant design. This unique aspect helps minimize bias related to implant variability and strengthens the validity of our comparative findings. However, limitations must be acknowledged. The follow‐up was limited to 5 years (with a median follow‐up of 27 months), precluding analysis of longer‐term complications such as wear or late aseptic loosening. Although the analysis was based on 1505 patients, a substantial sample size, it is important to note that only 28% of the initial cohort reached the 5‐year follow‐up, introducing a potential bias related to loss to follow‐up. Moreover, despite the use of adjusted models, some residual confounding may persist due to the nonrandomized nature of the study and surgeon‐dependent fixation choice, which was influenced by intraoperative assessment and preference. Potential unmeasured confounders include factors such as surgeon experience or variations in intraoperative alignment, which were not captured in the database. Given the multicentre design and 20‐year inclusion period, changes in surgical techniques and peri‐operative management may have influenced outcomes. However, the consistent implant design and adjustment for the inclusion period likely helped mitigate this bias. The absence of a significant difference does not preclude the possibility of delayed divergence between groups over time, and further long‐term studies are needed to explore these possibilities.

## CONCLUSIONS

In this large multicentre cohort of primary TKA with the same deep‐dish mobile bearing design, 5‐year survivorship was equivalent between cementless and hybrid fixation, regardless of whether the femoral or tibial component was cemented. Reoperation rates and functional outcomes were also comparable, suggesting that whether the TKA is cementless or hybrid does not influence mid‐term outcomes.

## AUTHOR CONTRIBUTIONS

**Ophélie Manchec**: Data collection; study design; article writing. **Emilie Bérard**: Statistical analysis; manuscript review. **Alessandro Carrozzo**: Manuscript review. **Regis Pailhé**: Manuscript review. **Etienne Cavaignac**: Study design; manuscript review and final approval of the manuscript.

## CONFLICT OF INTEREST STATEMENT

EC: consultant for Arthrex, Amplitude and Biobank. The remaining authors declare no conflict of interest.

## ETHICS STATEMENT

The use of the database was conducted under the authorization of the CNIL, registered in CliniRecord under the N°1355265. Amplitude® has registered the data for the long‐term evaluation of the SCORE prosthesis on the public platform ‘Health Data Hub’ under number N° F20210913151920. All data used in this study are sourced from this registry, which we managed according to the CNIL standard methodology MR‐004.

## Data Availability

The datasets generated and analyzed during the current study are not publicly available due to confidentiality agreements but are available from the corresponding author on reasonable request.
